# Expanded National Institutes of Health Stroke Scale and posterior circulation-specific stroke scales help address assessment blind spots in posterior circulation ischemic stroke

**DOI:** 10.3389/fneur.2026.1812120

**Published:** 2026-05-19

**Authors:** Qi Han, Mei-Hua Han

**Affiliations:** 1 Department of Neurology, Yanbian University Hospital, Yanji, China; 2 Department of Internal Medicine II, Chengxiang District Hospital of Putian, Putian, Fujian, China

**Keywords:** brainstem infarction, cerebellar infarction, expanded National Institutes of Health Stroke Scale, National Institutes of Health Stroke Scale, posterior circulation stroke, posterior circulation stroke scales

## Abstract

The National Institutes of Health Stroke Scale is the most widely used bedside scale for assessing neurological deficit severity in acute stroke and guiding early clinical decision-making. However, it has important limitations in posterior circulation stroke, particularly in cases involving the brainstem and cerebellum. Because posterior circulation ischemic stroke often presents with vertigo, nystagmus, truncal ataxia, bulbar dysfunction, and other cranial nerve-related symptoms, the conventional National Institutes of Health Stroke Scale may underestimate the initial severity of neurological impairment. This under-recognition can delay escalation of diagnostic evaluation and compromise prognostic stratification. This review specifically focuses on posterior circulation ischemic stroke because currently available posterior circulation-oriented derivatives of the National Institutes of Health Stroke Scale have been developed and clinically evaluated mainly in ischemic stroke cohorts. Recent studies suggest that the expanded National Institutes of Health Stroke Scale improves sensitivity to posterior circulation deficits missed by the conventional scale, whereas posterior circulation-specific tools such as the Posterior National Institutes of Health Stroke Scale and the Peoria Posterior Fossa Stroke Scale may further improve structured bedside assessment and prognostic stratification. Nevertheless, the evidence base remains limited by the small number of validation studies, lack of large multicenter external validation, and incomplete standardization of item definitions and scoring procedures. Future studies should focus on rigorous validation, standardization, and integration of these scales with imaging and clinical workflow to improve recognition and risk assessment in posterior circulation ischemic stroke.

## 1 Introduction

Since its introduction, the National Institutes of Health Stroke Scale (NIHSS) has established itself as an indispensable tool for assessing the severity of neurological deficits in patients with acute ischemic stroke and guiding clinical treatment decisions. It has been widely incorporated into both single-center and multicenter studies, earning recognition as the “gold standard” for stroke patient evaluation. Nevertheless, while the NIHSS demonstrates robust performance in assessing anterior circulation stroke (ACS), its limitations become increasingly prominent when applied to posterior circulation stroke (PCS) (1). PCS involves infarction within the territory supplied by the vertebrobasilar arterial system, representing 17–25% of all stroke cases (2). Intracranially, this vascular system is divided into proximal, middle, and distal segments. Embolic events account for 40–54% of PCS cases, making them the most prevalent etiology (3). Common clinical manifestations of PCS include dizziness, hemiplegia, dysarthria, ataxia, nystagmus, headache, and nausea/vomiting. Owing to its unique anatomical location and clinical presentation, PCS is often inadequately captured by the NIHSS, giving rise to the so-called “NIHSS blind spot.” This underestimation tends to result in lower NIHSS scores at admission, which may compromise early diagnosis, optimal treatment stratification, and accurate prognosis prediction. In recent years, researchers have proposed various strategies to address the NIHSS limitations in PCS assessment, with the extended NIHSS (e-NIHSS) and Posterior Circulation Stroke Scale (PCSS) attracting considerable attention (4, 5). Based on the 11 core items of the NIHSS, the e-NIHSS integrates additional PCS-specific dimensions, including detailed cranial nerve examinations, vertigo, and balance disturbances. This modification significantly enhances the sensitivity and accuracy of PCS severity assessment, thereby facilitating refined treatment decisions and prognosis estimation. The PCSS is a dedicated scale designed for the detailed quantification of brainstem and cerebellar dysfunction. Theoretically, it offers higher specificity, which aids in the early identification of PCS. However, its current clinical adoption remains limited due to multiple challenges, such as low awareness among clinicians, sluggish dissemination, high training costs, and difficulties in integrating into existing clinical workflows. Although the National Institutes of Health Stroke Scale is also used in intracerebral hemorrhage in contemporary practice, this review focuses on posterior circulation ischemic stroke because the currently available posterior circulation-oriented derivatives were primarily developed and clinically evaluated in ischemic stroke cohorts.

## 2 Limitations of the NIHSS in assessing posterior circulation stroke

The National Institutes of Health Stroke Scale (NIHSS) is a multi-item scoring tool developed to quantify neurological deficits in patients with stroke. Scores range from 0 to 42, with higher values indicating more severe neurological impairment. The scale covers multiple domains, including consciousness, eye movements, visual fields, facial palsy, motor function, sensation, ataxia, dysarthria, and aphasia. However, the NIHSS poses notable limitations when evaluating posterior circulation strokes.

### 2.1 Insufficient sensitivity to symptoms specific to posterior circulation stroke

The posterior circulation, particularly the brainstem and cerebellum, subserves distinct neurological functions such as balance, coordination, swallowing, speech, and ocular motility (e.g., nystagmus, diplopia). While certain NIHSS items—including those assessing motor function, sensation, and language—are highly sensitive to anterior circulation lesion-related symptoms (e.g., hemiplegia, aphasia), they either underweight or fail to account for manifestations typical of posterior circulation strokes, such as vertigo, ataxia, dysarthria, and cranial nerve palsies. For instance, the sole “ataxia” item, which partially reflects cerebellar function, carries low weighting. As a result, NIHSS scores often underestimate the true severity of neurological impairment in mild to moderate posterior circulation stroke. Patients with significant deficits may be inaccurately categorized as experiencing a “minor stroke” owing to low NIHSS scores (6, 7).

### 2.2 The “NIHSS blind spot” phenomenon

The “blind spot” in the NIHSS assessment denotes neurological deficits characteristic of posterior circulation stroke that are inadequately captured by the scale. This discrepancy can lead to a paradox wherein patients with severe posterior circulation infarction present with low NIHSS scores on admission. For example, among ischemic stroke patients with NIHSS scores ≤3, posterior circulation involvement correlates with higher disability rates—an association less prominent in those with scores of 4–5 (6). Consequently, such patients may be denied timely aggressive interventions, such as endovascular thrombectomy. Even among patients with low NIHSS scores (≤6), posterior circulation stroke remains linked to significant post-thrombectomy disability. Notably, studies report that up to 50% of these patients experience futile recanalization following endovascular thrombectomy, underscoring the NIHSS’s failure to fully delineate underlying severe neurological impairment (8).

### 2.3 Limitations of the NIHSS in prognostic prediction for stroke

Although the National Institutes of Health Stroke Scale (NIHSS) is widely utilized for predicting stroke outcomes, its predictive performance tends to be inferior in posterior circulation strokes (PCS) relative to anterior circulation strokes (ACS). PCS and ACS patients exhibit distinct clinical features, risk profiles, and outcome patterns (9). For example, a retrospective analysis demonstrated that PCS patients may have higher disability and mortality rates than ACS patients, despite presenting with lower NIHSS scores. This study further indicated that the NIHSS may insufficiently capture symptom severity in PCS. Additionally, younger patients evaluated via the NIHSS showed worse prognoses than older counterparts, whereas elderly patients had higher median NIHSS scores—findings that further underscore the limitations of the NIHSS in prognostic assessment (10). Moreover, an analysis of the RICAMIS trial (11) revealed substantial variability in functional outcomes even among patients with low NIHSS scores. Specifically, in the intervention group (12), 41.9% of patients with an NIHSS score of 6–8 achieved a modified Rankin Scale (mRS) score of 0–1, which was significantly higher than the 34.3% rate observed in the control group (13–15). These results confirm that the NIHSS score is not the sole determinant of stroke prognosis and highlight a potential “blind spot” in its ability to assess PCS (16).

## 3 Extended NIHSS and its application in posterior circulation stroke

To overcome the inherent limitations of the National Institutes of Health Stroke Scale (NIHSS) in assessing posterior circulation stroke (PCS), researchers have expanded the original NIHSS framework to establish the extended NIHSS (e-NIHSS) (17). By retaining the 11 core items of the NIHSS, the e-NIHSS integrates additional assessment indicators tailored to posterior circulation-specific neurological symptoms, thereby enabling more comprehensive capture of neurological deficits in PCS patients.

### 3.1 Composition and characteristics of e-NIHSS

The e-NIHSS incorporates posterior circulation-specific signs and symptoms, including vertical eye movement disturbances, nystagmus, Horner’s syndrome, glossopharyngeal nerve palsy, hypoglossal nerve palsy, and truncal ataxia. Tarnutzer et al. (18) demonstrated that the e-NIHSS exhibits excellent test–retest reliability and inter-rater reliability. In patients with PCS, e-NIHSS scores are, on average, 2 points higher than NIHSS scores; among the added items, nystagmus, Horner’s syndrome, and hypopharyngeal/hypoglossal nerve dysfunction show greater specificity compared with truncal ataxia and a positive Romberg sign. By enhancing the sensitivity of the NIHSS in evaluating neurological function in PCS, the e-NIHSS facilitates comprehensive and accurate assessment of patient conditions. It not only guides clinical treatment decisions and monitors disease progression as well as therapeutic responses but also confers partial prognostic value for clinical outcomes. Furthermore, it provides critical data support for various clinical trials targeting PCS.

### 3.2 Comparison of e-NIHSS and NIHSS

For the assessment of posterior circulation ischemic stroke (PCIS), the conventional NIHSS often underestimates typical posterior circulation deficits (e.g., oculomotor, vestibular/cerebellar, and bulbar signs) owing to its weighting bias toward anterior circulation and cortical functions (e.g., language and neglect). This results in a clinical discrepancy wherein low scores do not reliably reflect favorable outcomes. To address this limitation, Olivato et al. introduced the expanded NIHSS (e-NIHSS). This modification preserves the core structure of the NIHSS while incorporating posterior circulation-specific signs into existing items. Clinical comparative analyses have shown that in patients with posterior circulation infarction, the e-NIHSS score was, on average, approximately 2 points higher than the conventional NIHSS, with a statistically significant difference (*p* < 0.05), thereby demonstrating enhanced sensitivity in detecting posterior circulation deficits (19). Additionally, Roushdy et al. performed a longitudinal study of 79 patients with imaging-confirmed posterior circulation stroke. Compared with the NIHSS, the e-NIHSS more consistently assigned higher-grade severity stratification at both baseline and follow-up time points (e.g., median scores were 2 points higher at baseline and 24 h and 1 point higher at discharge; *p* < 0.001). When the e-NIHSS score exceeded that of the NIHSS, patients exhibited poorer 90-day functional outcomes, reflecting superior prognostic sensitivity. Their receiver operating characteristic (ROC) curve analysis revealed an area under the curve (AUC) of 0.858 for predicting 90-day adverse outcomes when the e-NIHSS cutoff was set at ≥8, with a sensitivity of 82% and specificity of 81% (20). This finding is consistent with previous large-scale evidence suggesting a lower threshold for the NIHSS in posterior circulation stroke. Schneider et al. (21) enrolled 372 patients with posterior circulation stroke and 1,197 with anterior circulation stroke and found that the median admission NIHSS score was only 2 in posterior circulation patients (vs. 7 in anterior circulation patients); 71% of posterior circulation patients had an NIHSS score of ≤4, yet 15% still experienced adverse 3-month outcomes. The optimal NIHSS cutoff value for outcome prediction was 4 in posterior circulation patients (vs. 8 in anterior circulation patients), indicating that exclusive reliance on the NIHSS may underestimate the risk of adverse outcomes in posterior circulation stroke. Collectively, current clinical evidence confirms that the e-NIHSS more accurately reflects the burden of posterior circulation-related deficits than the NIHSS in PCIS and may improve the stratification of 90-day functional outcomes. In emergency and stroke unit practice, the e-NIHSS can serve as a supplementary assessment tool for patients with suspected posterior circulation stroke, while the NIHSS remains the universal baseline scale—thus minimizing low-score misclassification.

An additional point that merits clarification is the behavior of the expanded National Institutes of Health Stroke Scale in anterior circulation stroke. Current evidence suggests that the clinical advantage of the expanded scale is concentrated mainly in posterior circulation ischemic stroke, because the added items target posterior fossa and brainstem manifestations that are usually absent in anterior circulation lesions. Accordingly, in typical anterior circulation stroke, the expanded scale would be expected to remain close to the conventional National Institutes of Health Stroke Scale, and there is currently no strong evidence showing a clinically meaningful systematic score increase comparable to that observed in posterior circulation stroke. In this sense, the expanded scale should be viewed primarily as a posterior circulation-sensitive complement rather than a universal replacement for the conventional National Institutes of Health Stroke Scale. The specific modifications to the e-NIHSS are detailed in Table 1.

**Table 1:** Key differences between NIHSS and e-NIHSS for posterior circulation ischemic stroke assessment.

Domain	NIHSS	e-NIHSS (detailed operational description)	Clinical implication
Vertical gaze	No dedicated vertical gaze item	0 = normal; 1 = partial limitation of vertical gaze; 2 = complete vertical gaze palsy	Better captures dorsal midbrain/brainstem ocular motor dysfunction
Nystagmus	Not directly scored	0 = absent; 1 = horizontal nystagmus; 2 = vertical or rotatory nystagmus	Better captures vestibulo-ocular/posterior fossa signs
Horner syndrome	Not explicitly scored	0 = absent; 1 = present	Reflects brainstem sympathetic pathway involvement
Glossopharyngeal/bulbar function	No dedicated CN IX/X item	0 = absent; 1 = dysarthria/hoarseness without clear swallowing compromise; 2 = dysphagia or marked bulbar dysfunction	Improves assessment of aspiration-relevant bulbar signs
Hypoglossal function	No dedicated CN XII item	0 = absent; 1 = mild tongue weakness/deviation; 2 = severe tongue paresis affecting articulation or swallowing	Captures lower cranial nerve deficit not represented in NIHSS
Romberg sign	Not included	0 = negative; 1 = positive	Detects balance impairment not represented in limb ataxia scoring

Comparison between the conventional National Institutes of Health Stroke Scale and the expanded National Institutes of Health Stroke Scale, emphasizing additional posterior circulation-oriented items and their operational score ranges. To improve interpretability, the expanded scale items are shown with bedside-oriented scoring definitions for mild versus severe abnormality, particularly for vertical gaze limitation, nystagmus, bulbar dysfunction, and truncal ataxia.

CN, cranial nerve; NIHSS, National Institutes of Health Stroke Scale; PCIS, posterior circulation ischemic stroke.

### 3.3 Clinical significance of e-NIHSS

The clinical significance of e-NIHSS primarily lies in its reinforcement of the “underdiagnosed and underestimated” aspects of posterior circulation ischemic stroke. Compared to the NIHSS, which focuses primarily on cortical function and limb movement, e-NIHSS incorporates key posterior circulation signs such as vertical gaze, nystagmus, Horner’s syndrome, lower cranial nerve function, Romberg’s sign, and trunk ataxia. This ensures that patients with mild or atypical presentations are no longer underestimated due to low NIHSS scores (18). Consequently, it heightens vigilance for posterior circulation strokes during emergency triage and early stroke unit assessment, reducing the risk of missed or misdiagnosed cases. More precise baseline quantification also facilitates refined treatment decisions: When NIHSS is low but e-NIHSS is significantly elevated, it suggests potential overlooked brainstem/cerebellar involvement. Clinicians can then proactively enhance imaging and vascular assessments, reevaluating the indications and risk–benefit ratios for intravenous thrombolysis or endovascular therapy. Simultaneously, e-NIHSS’s inclusion of swallowing, articulation, and bulbar palsy information provides greater directionality for determining monitoring levels, airway management, and nutritional strategies (20). Regarding prognostic assessment, e-NIHSS offers a more comprehensive spectrum of posterior circulation deficits, potentially enhancing the ability to stratify functional outcomes and complication risks while providing more sensitive quantitative indicators for treatment follow-up. Finally, from a research and management perspective, the adoption of e-NIHSS helps standardize the assessment of posterior circulation strokes, improves data comparability across centers, reduces measurement errors like “same disease, different scores,” and lays the groundwork for multicenter studies and the accumulation of real-world evidence (21).

## 4 Assessment scales for posterior circulation stroke and their evolution

Beyond the extensions made within the framework of the National Institutes of Health Stroke Scale (NIHSS)—such as the extended NIHSS (e-NIHSS)—researchers have strived in recent years to develop assessment tools with a heightened focus on posterior circulation/posterior fossa stroke. Representative advances include the Peoria Posterior Fossa Stroke Scale (PPFSS) and the Posterior NIHSS (POST-NIHSS). The shared rationale underpinning these scales lies in the limitation that the conventional NIHSS inadequately captures common posterior circulation-related signs, including brainstem ocular motor deficits, vestibular/cerebellar manifestations, and bulbar palsy-associated symptoms. This deficiency may lead to a clinical mismatch characterized by low NIHSS scores but non-negligible clinical risks, thereby undermining the accuracy of early risk stratification and prognostic judgment in posterior circulation stroke patients (22).

### 4.1 Composition and characteristics of posterior circulation-focused scales (illustrated by PPFSS and POST-NIHSS)

Centered on the clinical signs of posterior fossa syndrome, the PPFSS establishes a structured bedside assessment framework encompassing five core domains: ocular motility/nystagmus, vestibular function tests (e.g., head impulse test), auditory function, pharyngeal/bulbar function, and balance. Its primary objective is to address the insufficient documentation of posterior fossa signs inherent in the NIHSS. It is critical to emphasize that the PPFSS remains in the conceptual/descriptive phase to date. Its diagnostic efficacy, inter-rater reliability, and external validity have not yet been validated by rigorous clinical studies; thus, it is more appropriately employed as a supplementary bedside examination framework rather than a standalone alternative to existing scales (23). In contrast, the POST-NIHSS adopts a weighted compensation strategy based on the original NIHSS. It enhances the NIHSS’s prognostic stratification capacity for posterior circulation stroke by adding weighted scores for key deficit items closely correlated with poor outcomes—including ataxia, dysphagia, and abnormal cough reflex. A cohort study of posterior circulation stroke patients conducted by Alemseged et al. demonstrated that the POST-NIHSS yields superior prognostic predictive accuracy compared with the conventional NIHSS. Notably, it facilitates the identification of hidden high-risk subgroups among patients with an NIHSS score < 10 (24).

### 4.2 Comparison with the NIHSS: divergent design pathways and evidence disparities

Unlike the e-NIHSS, which expands posterior circulation-related signs within the existing NIHSS item set, the PPFSS represents a paradigm of sign domain reorganization grounded in posterior fossa syndrome phenotypes, emphasizing systematic documentation of posterior circulation-specific features. In contrast, the POST-NIHSS preserves the core structure of the NIHSS and improves prognostic prediction by integrating weighted scores for a limited number of critical deficit items. In terms of evidence strength, the POST-NIHSS is supported by cohort-based validation studies confirming its added value in prognostic stratification. By comparison, the PPFSS currently serves primarily as a practical bedside assessment tool, and its clinical translation requires further external validation and feasibility evaluation. A detailed comparison of the three scales is provided in Table 2.

**Table 2:** Comparison of NIHSS, PPFSS, and POST-NIHSS.

Domain	NIHSS	PPFSS	POST-NIHSS
Focus	General deficit scale; acute stroke severity (30–32)	Posterior fossa symptom domains underweighted in NIHSS (5)	Posterior circulation prognostic stratification (NIHSS-based) (25, 33)
Scoring	11 items; 0–42 (32)	6 domains; 0–15 (Eyes, Nystagmus, Head impulse, Hearing, Pharynx, Balance) (34)	NIHSS + posterior-weighted add-ons (max +12) (33)
Calculation (reproducible)	Official NIHSS sum (32)	Sum of published domain scores (34)	POST-NIHSS = NIHSS +3 (gait/truncal ataxia) + 4 (dysphagia) + 5 (abnormal/weak voluntary cough) (33)
Posterior signs	Limited vestibular/ocular-motor/bulbar/truncal-gait capture; low scores may occur in PCIS (25)	Explicit ocular-motor + vestibular surrogate + bulbar + balance/gait documentation (34)	Adds bulbar and ataxia/cough weights to address NIHSS underweighting in PCIS (33)
Typical use	Baseline scale across settings (32)	Adjunct when PCIS suspected and NIHSS seems low vs. symptoms (34)	Outcome risk stratification in PCIS (often NIHSS <10) (33)
Strengths	Standardized; widely validated; cross-study comparability (30–32)	High face validity for posterior fossa syndromes; structured bedside record (34)	Additive (no NIHSS redesign); improved outcome prediction vs. NIHSS in cohorts (33)
Key limitations	May underestimate PCIS; does not replace targeted posterior exam (25)	Evidence mainly from initial report; broader prospective reliability/utility studies needed; examiner skill required (e.g., head impulse) (34)	Dysphagia/cough assessment may be workflow-limited; external validation in diverse settings needed (33)

Condensed Comparison for Manuscript Submission. The National Institutes of Health Stroke Scale (NIHSS) is a widely validated clinical instrument for quantifying acute stroke-associated neurological deficits; the Peoria Posterior Fossa Stroke Scale (PPFSS) is a bedside assessment tool focusing exclusively on posterior fossa-related neurological domains; and the Posterior NIH Stroke Scale (POST-NIHSS) represents an NIHSS-derived prognostic adjunct optimized for posterior circulation stroke. All citations herein correspond to the reference list provided above.

NIHSS, National Institutes of Health Stroke Scale; PPFSS, Peoria Posterior Fossa Stroke Scale; POST-NIHSS, Posterior NIH Stroke Scale; PCIS, posterior circulation ischemic stroke.

### 4.3 Clinical significance and challenges of posterior circulation stroke assessment scales

The core value of disease-specific or posterior circulation-weighted scales [e.g., Posterior Circulation-Focused Stroke Scale (PPFSS), Posterior Stroke National Institutes of Health Stroke Scale (POST-NIHSS)] lies in the structured compensation for the spectrum of posterior fossa deficits that are prone to underassessment by the standard NIHSS (25, 26). These scales typically prioritize quantitative documentation of signs associated with oculomotor/vestibular dysfunction, cerebellar ataxia, and bulbar palsy. Theoretically, this design enhances the detection sensitivity for mild and atypical posterior circulation strokes, mitigates the misjudgment of “low score indicating low risk,” and thus facilitates the early identification of high-risk individuals across the prehospital-emergency-stroke unit triage continuum. Such timely stratification further gains valuable time for decision-making regarding imaging protocols, monitoring intensity, swallowing management, and reperfusion therapy eligibility (27). Furthermore, with adequate validation and the establishment of standardized operational procedures, these scales hold the potential to evolve into reproducible, standardized outcome measures for posterior circulation stroke research, thereby improving the comparability of multicenter data and the consistency of collaborative studies. Nevertheless, the primary challenges at present stem from the incompleteness of the evidence chain. External validation, inter-rater reliability, sensitivity to treatment responses, and real-world feasibility of these scales still require support from large-sample, prospective, multi-setting studies (28, 29). The assessment of certain signs (e.g., vestibular/oculomotor examinations, swallowing and cough evaluations) is highly dependent on the operator’s clinical experience and procedural conditions, which inevitably introduces variability in training costs and implementation practices. In addition, a critical hurdle to translating these scales from “conceptual optimization” to “widely applicable tools” is to achieve seamless integration with existing NIHSS workflows, imaging pathways, and stroke green channels—without imposing a significant additional burden on emergency department workloads. Concurrently, it is imperative to define rational triage thresholds and clinical trigger strategies to guide their practical application.

## 5 e-NIHSS and posterior circulation-focused scales address limitations of NIHSS in posterior circulation cerebral infarction assessment

The National Institutes of Health Stroke Scale (NIHSS) is a widely used tool for assessing neurological deficits related to acute stroke. Its item configurations and weightings are more in line with anterior circulation and cortical functional phenotypes. Consequently, it is insufficient in capturing brainstem-vestibular-cerebellar deficits prevalent in posterior circulation ischemic stroke (PCIS), such as vertical gaze palsy, nystagmus, Horner’s syndrome, bulbar palsy-related dysphagia/abnormal coughing, and trunk/gait ataxia. Clinically, this limitation translates to systematic risk underestimation: patients may present with low NIHSS scores yet retain considerable clinical risk, undermining the consistency of early risk stratification, monitoring protocols, and prognostic evaluation. The extended NIHSS (e-NIHSS) preserves the core framework of the original NIHSS while incorporating key items, including vertical gaze function, nystagmus, Horner’s syndrome, lower cranial nerve function, and trunk ataxia. This modification enhances alignment with posterior circulation deficit profiles, improves detection of mild or atypical PCIS, and partially optimizes stratification of functional outcomes. Among scales specifically designed for posterior circulation assessment, POST-NIHSS adopts a “weighted compensation” approach: on the basis of baseline NIHSS scores, it incorporates bonus points for prognostically relevant posterior circulation factors (e.g., ataxia, dysphagia, abnormal cough) to strengthen prognostic risk identification in PCIS. The Posterior Fossa Stroke Scale (PPFSS), by contrast, relies primarily on structured assessments of bedside signs, with an emphasis on standardized documentation of key posterior fossa syndrome manifestations. It is well-suited for supplementary assessment in cases where NIHSS scores are low but clinical suspicion for posterior circulation lesions remains high. Collectively, e-NIHSS and posterior circulation-focused scales (PCFSs) share core values: mitigating the underestimation of posterior circulation deficits by the NIHSS, improving identification of “low NIHSS but high-risk” patients, and thereby enabling more tailored selection of imaging pathways, swallowing management strategies, and surveillance stratification.

**Figure 1:**
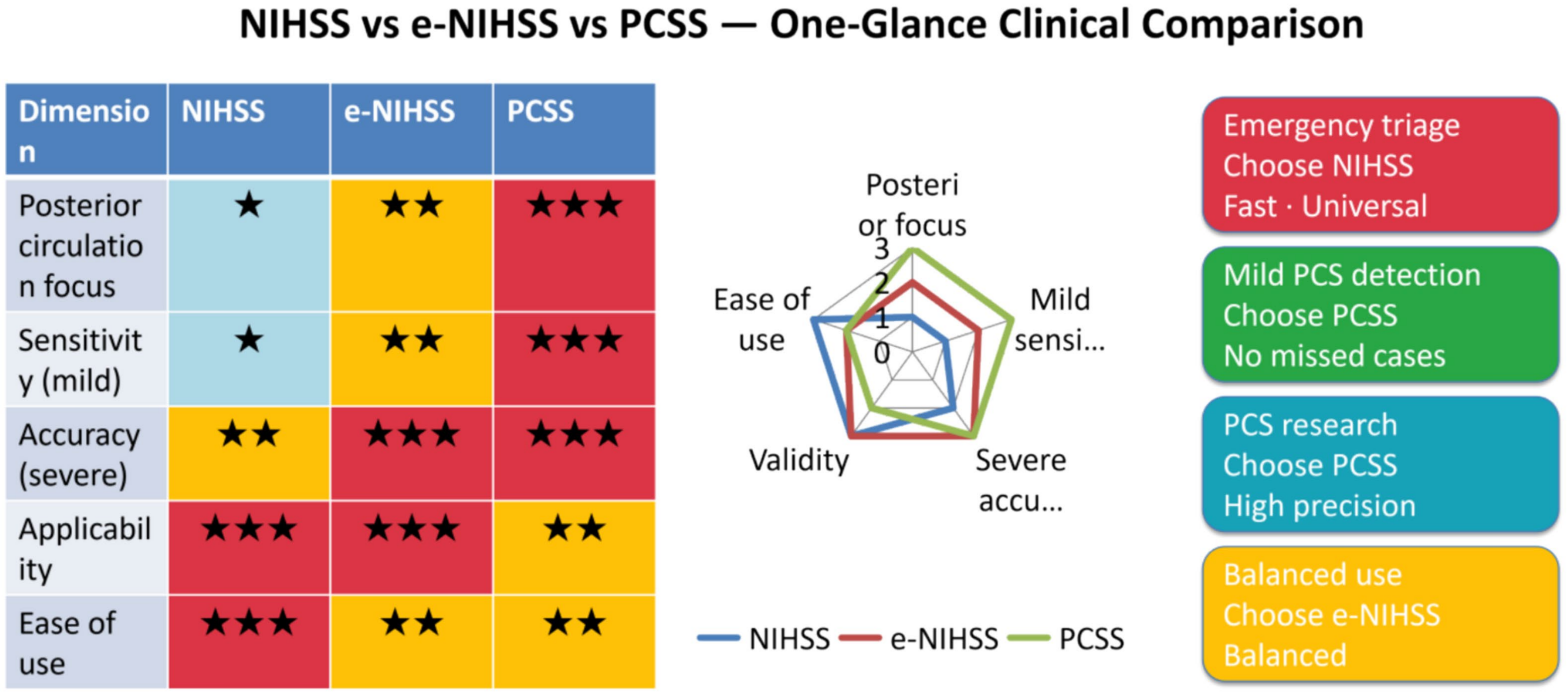
One-glance comparison of NIHSS, e-NIHSS, and PCSS.

To allow readers to quickly compare the capability structures of the three scales, radar charts were used for visual presentation in this study. Notably, the 1–3 point scale in the radar chart is not derived from raw data fitting of a single cohort but rather based on a predefined semi-quantitative scoring framework that structurally maps the dimensions of “scale design coverage—evidence support—implementability.” Scores for each dimension were jointly determined by three factors: the coverage of scale items relative to design objectives, conclusions from published comparative or validation studies, and feasibility in clinical workflows. To ensure the traceability and auditability of scoring outcomes, we present the relevant scoring adjudication criteria and corresponding literature citations in full detail (Table 3). To mitigate subjective scoring bias and improve methodological robustness, two investigators with experience in stroke assessment independently conducted scoring. Consistency metrics were calculated, and discrepancies were resolved via a pre-specified consensus process. Sensitivity analyses were performed to confirm that adjustments to individual scores do not substantially alter the overall “capability profile” or the direction of conclusions derived from the radar chart. With these transparency and robustness controls in place, the radar chart serves as an intuitive comparative tool for clinicians and readers, illustrating the relative strengths and trade-offs of different scales across posterior circulation-related dimensions. This scoring methodology was used to generate the comparative charts, highlighting the relative advantages and limitations of the three scales—importantly, it is not intended to replace quantitative inferences regarding real-world diagnostic performance or prognostic models (Figure 1).

**Table 3:** Scoring criteria and literature-based evidence for radar chart comparison (1–3 scale).

Domain (radar axis)	NIHSS (score; key evidence)	e-NIHSS (score; key evidence)	Posterior-circulation–weighted scale (POST-NIHSS) (score; key evidence)
Posterior circulation focus	1; NIHSS is anterior-weighted and may underestimate posterior circulation deficits (35, 36)	2; Expanded NIHSS increases posterior-circulation sensitivity via additional posterior signs (37)	3; Posterior-specific weighting validated for prognostication in posterior circulation stroke (38)
Sensitivity (mild)	1; Low NIHSS does not exclude stroke; mild posterior presentations can be missed (35, 36)	2; Improved vs. NIHSS, but still a general framework (37)	3; Improves risk stratification among mild–moderate posterior circulation strokes (38)
Accuracy (severe)	2; Captures global severity but remains less aligned with posterior-fossa symptom burden (35, 36)	3; Expanded posterior items enhance assessment vs. NIHSS (37)	3; Posterior-weighted approach improves prognostic accuracy in posterior circulation stroke (38)
Validity (reliability/validation)	3; Original development demonstrates inter-rater and test–retest reliability; widely used (39, 40)	2–3; Published extension; smaller evidence base than NIHSS but peer-reviewed (37)	2–3; Peer-reviewed and validated; broader external validation is evolving vs. NIHSS (38)
Ease of use	3; Rapid standardized bedside assessment with mature training pathways (39, 40)	2; Added items increase complexity/training needs (37)	2; Posterior-focused signs (e.g., bulbar/cerebellar) require more specialized expertise (38, 41)

Scale: 1 = low/limited; 2 = moderate; 3 = high/optimal.

This figure presents a comparative analysis of the National Institutes of Health Stroke Scale (NIHSS), electronic NIHSS (e-NIHSS), and Posterior Circulation Stroke Scale (PCSS) across core clinical domains, integrating three complementary components: a simplified tabular summary, a radar chart, and scenario-specific clinical recommendations. The tabular summary quantifies critical assessment attributes—including posterior circulation specificity, sensitivity for subtle neurological deficits, diagnostic accuracy in severe stroke, real-world clinical applicability, and ease of administration—via a standardized color- and symbol-coded annotation system. The accompanying radar chart (on a 1–3 ordinal scale) delineates the performance spectra of these three tools, accentuating PCSS’s unique superiority in posterior circulation lesion evaluation, e-NIHSS’s well-rounded performance across diverse clinical scenarios, and the inherent operational simplicity of the original NIHSS.

## 6 Conclusion

The conventional National Institutes of Health Stroke Scale remains the standard bedside tool for acute stroke assessment, but it has a well-recognized blind spot in posterior circulation ischemic stroke because many brainstem, vestibular, bulbar, and truncal cerebellar signs are insufficiently represented. Current evidence indicates that the expanded National Institutes of Health Stroke Scale improves bedside sensitivity to posterior circulation deficits, whereas the Posterior National Institutes of Health Stroke Scale strengthens prognostic stratification in posterior circulation stroke. The Peoria Posterior Fossa Stroke Scale is conceptually valuable as a structured posterior fossa examination framework, but its clinical role still requires broader validation. At present, these scales should be considered complementary rather than replacement tools for the conventional National Institutes of Health Stroke Scale. Future work should prioritize multicenter external validation, standardized item definitions, and integration with imaging and workflow-based triage strategies to improve the recognition and risk stratification of posterior circulation ischemic stroke.
